# Supplementary material for: Identification and characterization of a new isoform of small GTPase RhoE
Source: Commun Biol. 2020 Oct 15;3:572. doi: 10.1038/s42003-020-01295-4 (PMC7562701; doi:10.1038/s42003-020-01295-4)
Supplement: Supplementary file 1 — Supplementary Information [file 42003_2020_1295_MOESM1_ESM.pdf]

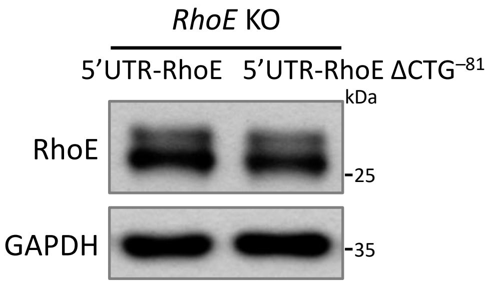

Supplement Figure 1. Predicted aTIS in *RhoE* 5'UTR is not responsible for new RhoE isoform expression. *RhoE* knockout (*RhoE* KO) HeLa cells were transfected with the indicated expression plasmids and cell lysates were immunoblotted for RhoE.

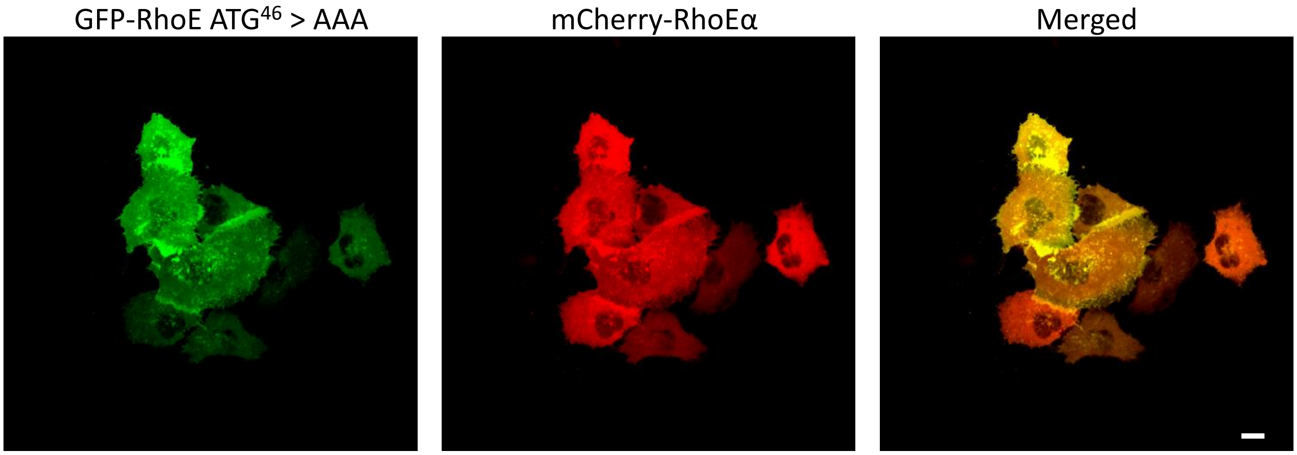

Supplement Figure 2. Confocal images exhibited subcellular localizations of GFP-RhoE with ATG[46] to AAA mutation and mCherry-RhoEα in HeLa cells. Scale bar: 10 μm.

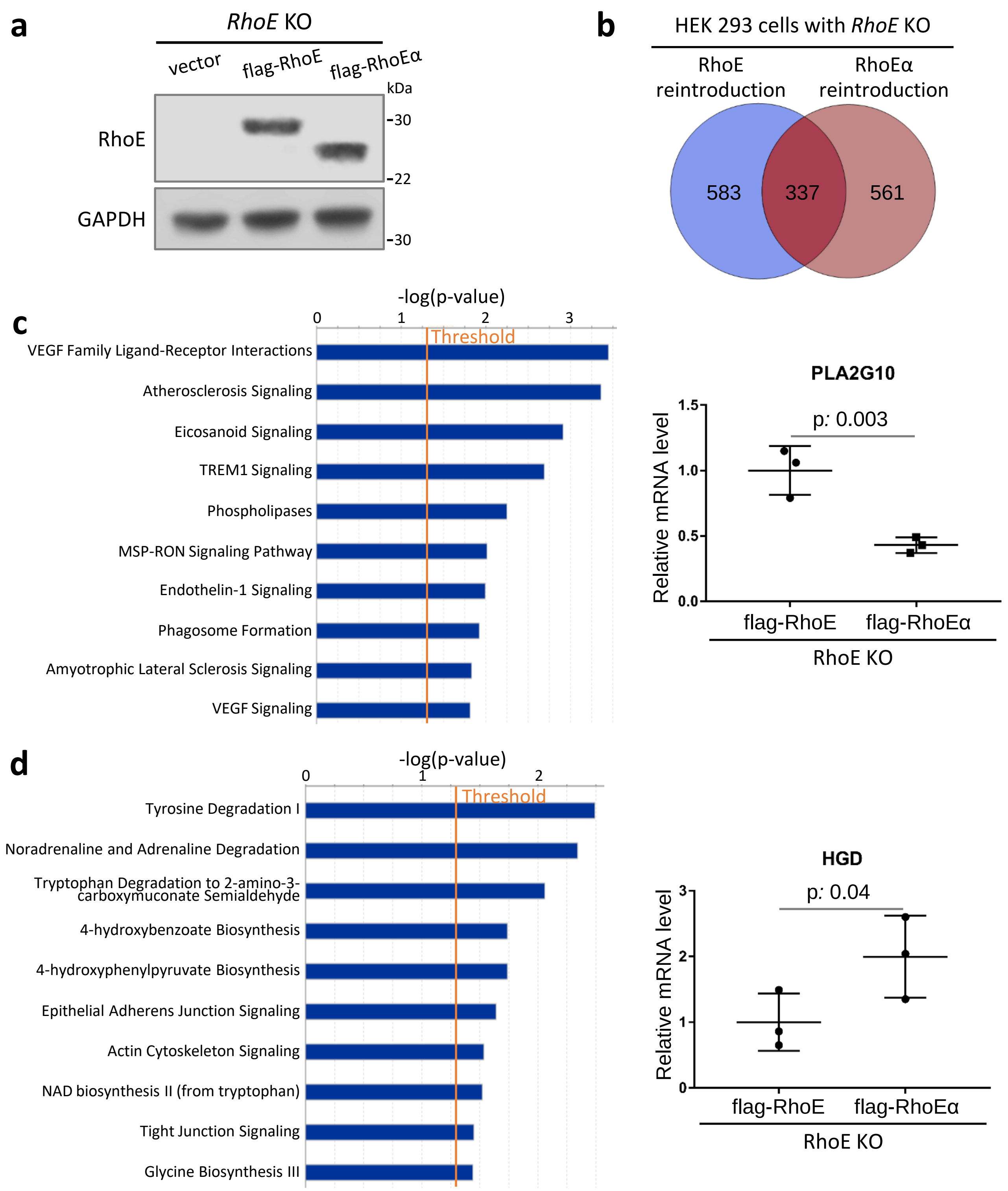

Supplement Figure 3. Transcriptomes differentially regulated by RhoE and RhoEα in HEK 293 cells. HEK 293 cells with *RhoE* knockout (*RhoE* KO) were transfected with flag-RhoE or flag-RhoEα expression plasmid individually. **a** Indicated expression plasmids were validated by immunoblot analysis in the transfected RhoE KO HEK 293 cells. **b** RNA-Seq was performed before and after transfection. RhoE re-introduction led to 920 genes change > 2-fold and RhoEα re-introduction led to 898 genes change > 2-fold. 337 genes were overlapped between two groups. **c** IPA analysis of the 583 genes regulated only by RhoE, and **d** the 561 genes regulated only by RhoEα. Differential expression levels of genes in top changed pathway were validated by RT-qPCR. PLA2G10 and HGD were chosen for the assessment (right panels of **c** and **d**). n =3.

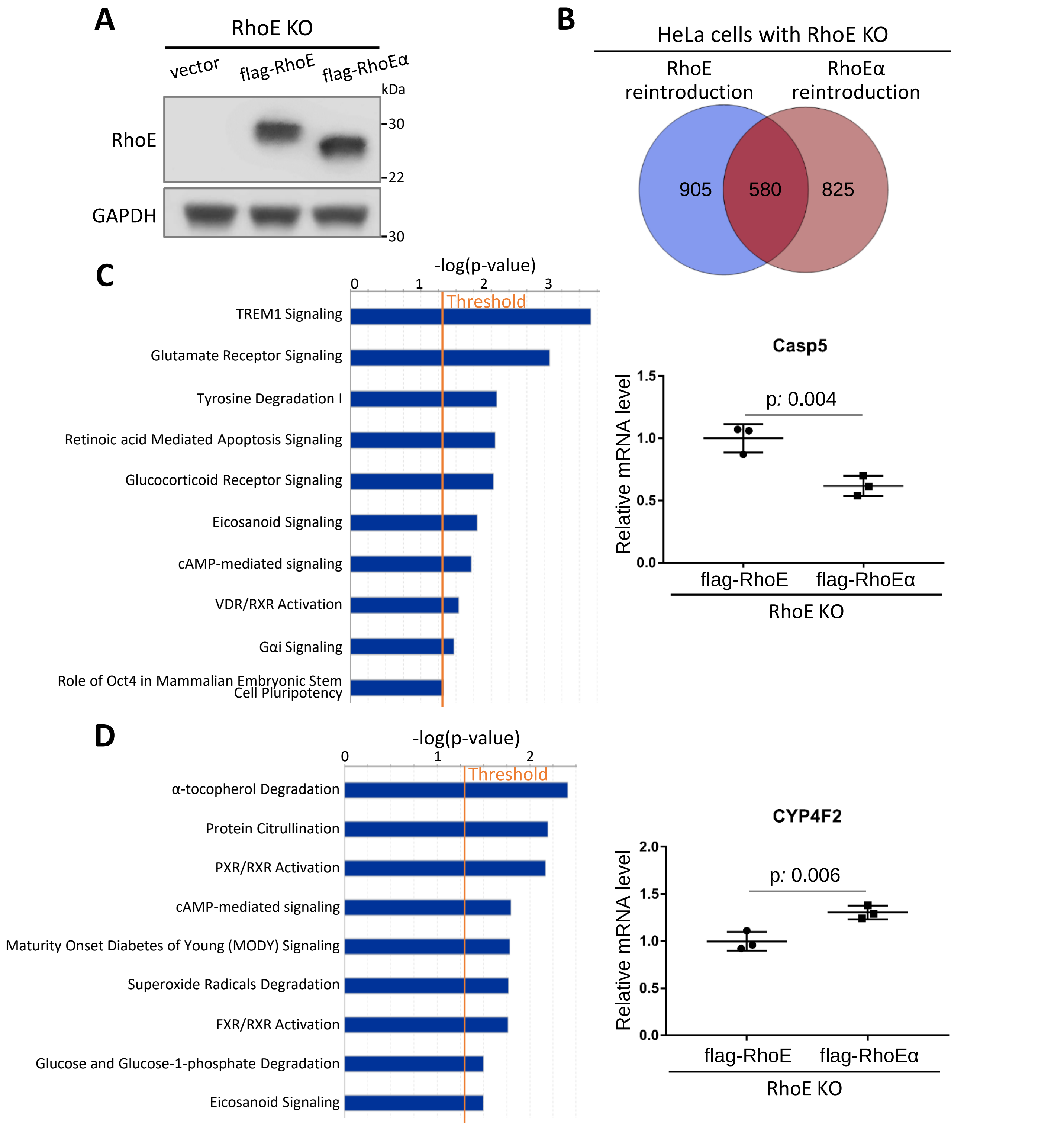

Supplement Figure 4. Transcriptomes differentially regulated by RhoE and RhoEα in HeLa cells. HeLa cells with *RhoE* knockout (RhoE KO) were transfected with flag-RhoE or flag-RhoEα plasmid individually. **a** Indicated expression plasmids were validated by immunoblot analysis in the transfected RhoE KO HeLa cells. **b** RNA-Seq was performed before and after transfection. RhoE re-introduction led to 1485 genes change > 2-fold and RhoEα re-introduction led to 1405 genes change > 2-fold. 580 genes were overlapped between two groups. **c** IPA analysis of the 905 genes regulated only by RhoE, and **d** the 825 genes regulated only by RhoEα. Differential expression levels of genes in top changed pathway were validated by RT-qPCR. Casp5 and CYP4F2 were chosen for the assessment (right panels of **c** and **d**). n =3.

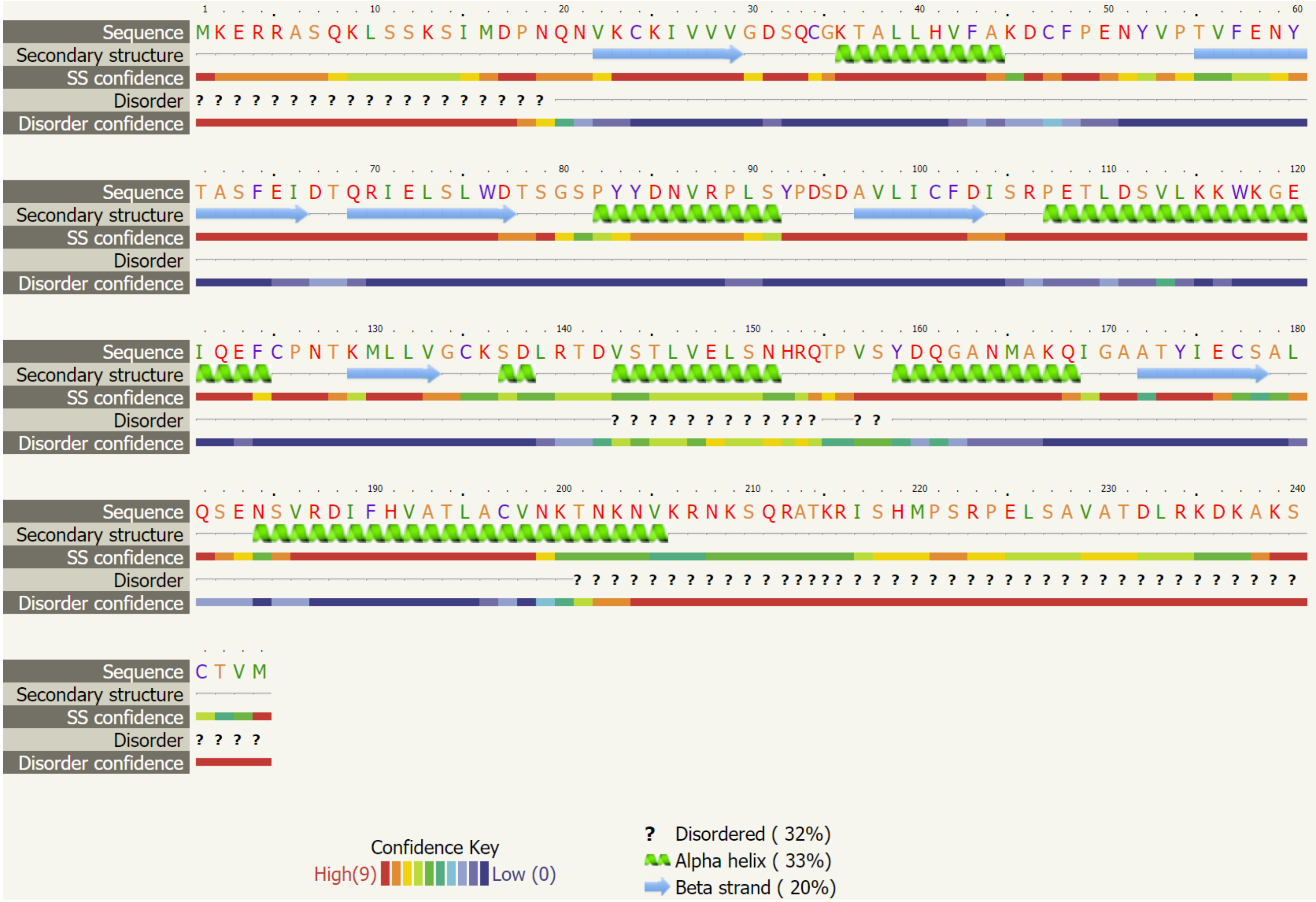

Supplement Figure 5. RhoE protein secondary structure analysis. RhoE full length was assessed in the Phyre2 web portal and no secondary structures were suggested within the first 21 amino acids of RhoE.

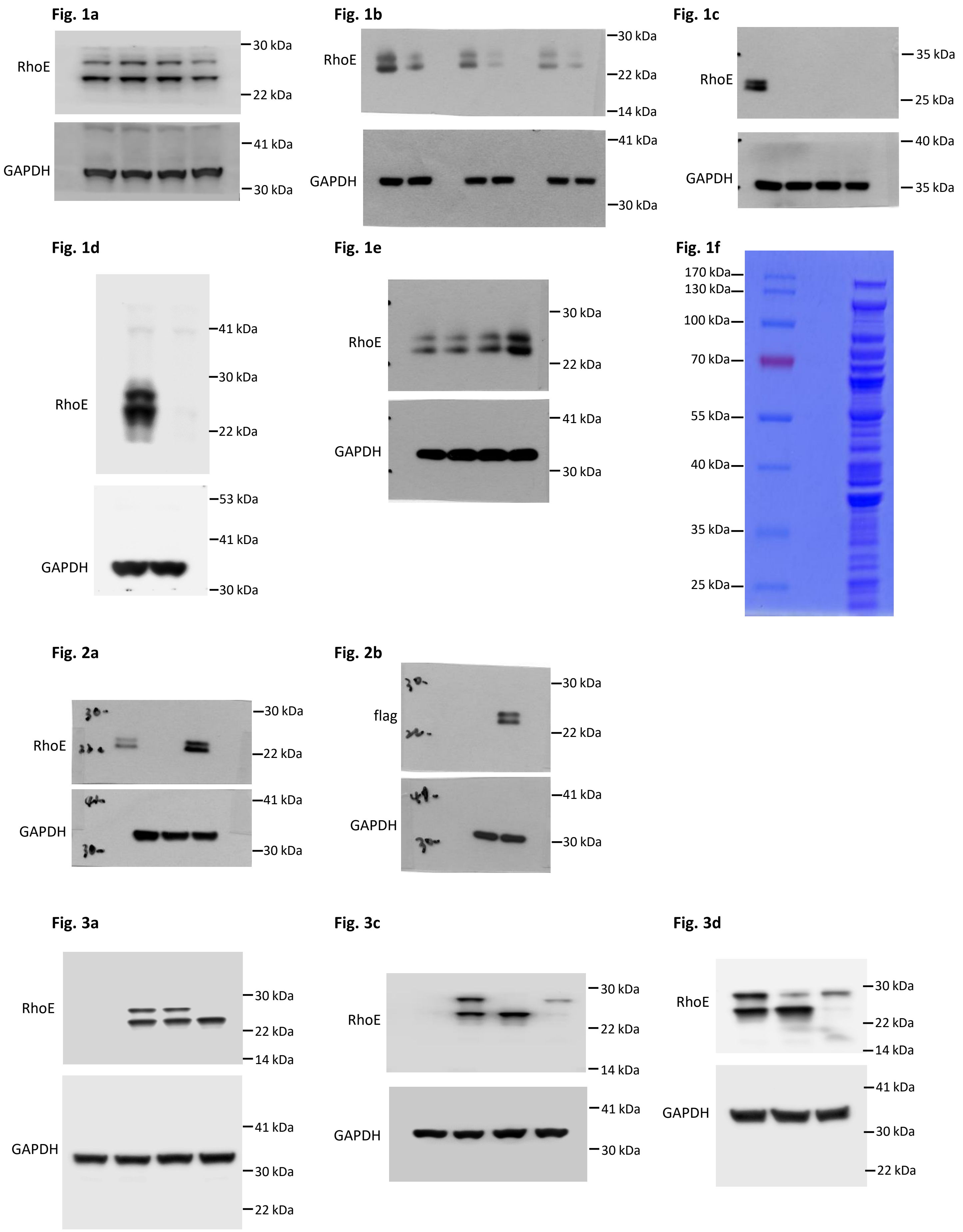

Supplement Figure 6. Uncropped immunoblot images and coomassie blue-stained gel for Figures 1-3.

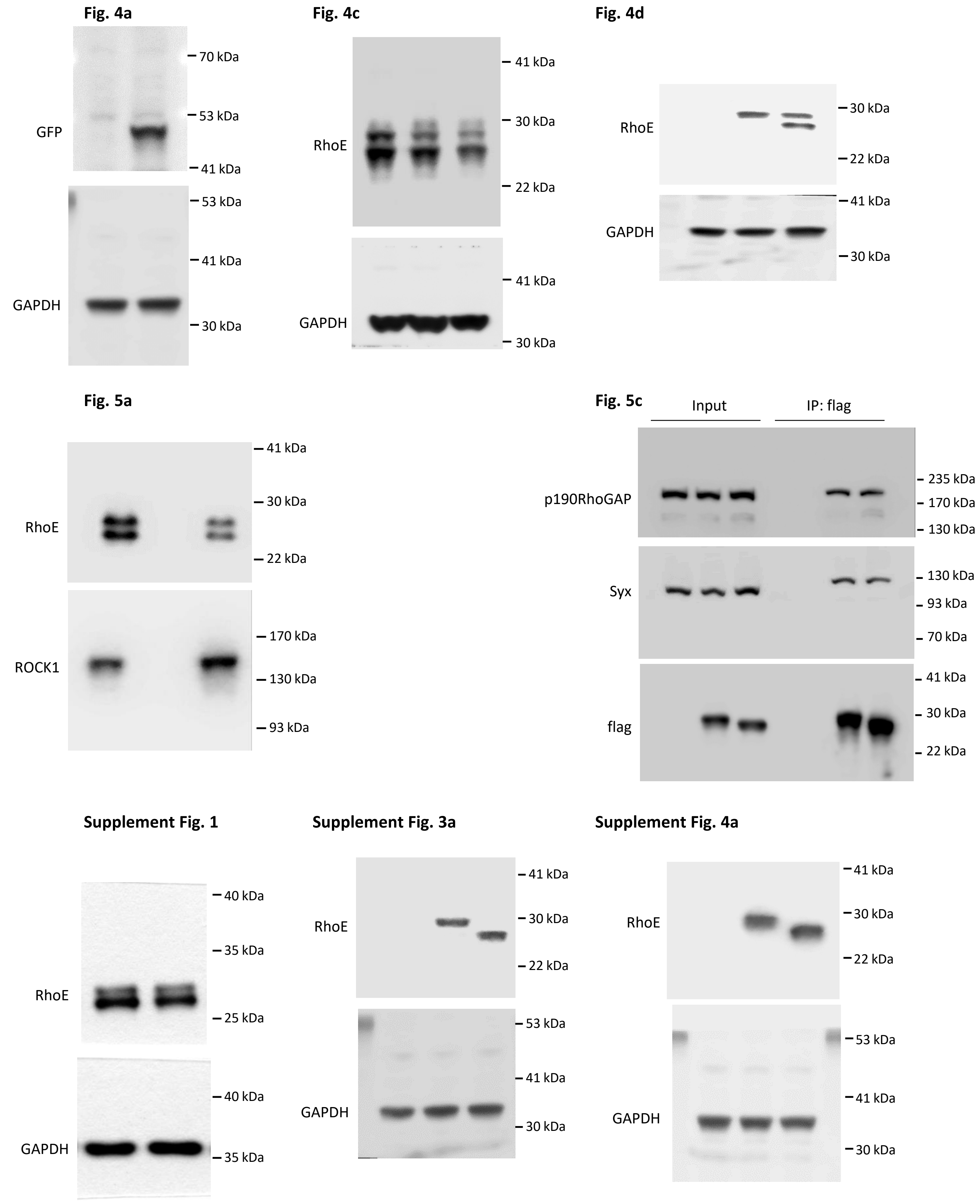

Supplement Figure 7. Uncropped immunoblot images for Figure 4-5 and Supplement Figures 1, 3 and 4.
